# The Effect of Secure Attachment State and Infant Facial Expressions on Childless Adults’ Parental Motivation

**DOI:** 10.3389/fpsyg.2016.01237

**Published:** 2016-08-17

**Authors:** Fangyuan Ding, Dajun Zhang, Gang Cheng

**Affiliations:** ^1^Faculty of Psychology, Southwest UniversityChongqing, China; ^2^Center for Mental Health Education, Southwest UniversityChongqing, China; ^3^School of Educational Science, Guizhou Normal UniversityGuiyang, China

**Keywords:** secure adult attachment state, parental motivation, infant facial expression, liking, wanting

## Abstract

This study examined the association between infant facial expressions and parental motivation as well as the interaction between attachment state and expressions. Two-hundred eighteen childless adults (*M*_age_ = 19.22, 118 males, 100 females) were recruited. Participants completed the Chinese version of the State Adult Attachment Measure and the E-prime test, which comprised three components (a) liking, the specific hedonic experience in reaction to laughing, neutral, and crying infant faces; (b) representational responding, actively seeking infant faces with specific expressions; and (c) evoked responding, actively retaining images of three different infant facial expressions. While the first component refers to the “liking” of infants, the second and third components entail the “wanting” of an infant. Random intercepts multilevel models with emotion nested within participants revealed a significant interaction between secure attachment state and emotion on both liking and representational response. A hierarchical regression analysis was conducted to examine the unique contributions of secure attachment state. Findings demonstrated that, after controlling for sex, anxious, and avoidant, secure attachment state positively predicted parental motivations (liking and wanting) in the neutral and crying conditions, but not the laughing condition. These findings demonstrate the significant role of secure attachment state in parental motivation, specifically when infants display uncertain and negative emotions.

## Introduction

Attachment relationships between infants and their caregivers influence the infants’ rate of survival as well as its’ social, emotional, and cognitive development (Insel and Young, 2001; Sroufe et al., 2005). Based on attachment theory, Mikulincer et al. (2005) proposed that what Bowlby (1969) referred to as the “caregiving behavioral system,” is an inherent behavioral system that responds to the needs of dependent others, particularly children. Parents’ caregiving behavior system is reported by Bowlby (1969, 2008) as complementary to the child’s attachment behavior system, and the principal function is to protect one’s child from danger. Therefore, in healthy parent–child relationships, the child’s attachment system and the parents’ caregiving system must interact to serve the common function of protection and survival of offspring.

With regard to how the caregiving behavioral system is influenced by the attachment system, Mikulincer et al. (2005) suggested that secure attachment activates the caregiving system indirectly and instead provides a stable psychological foundation that others’ suffering or the interdependence entailed by caregiving cannot prevail over. In other words, perceived needs for self-protection are lessened by the sense of attachment security (Mikulincer and Shaver, 2005); consequently, secure attachment allows individuals to shift their energy to the caregiving behavioral system (Mikulincer et al., 2002).

A large body of research has explored the link between attachment style in adulthood (e.g., self-reported and interview studies) and parental aspects. Studies have consistently shown that across three domains of parenting (i.e., cognition, emotion, and behavior) secure attachment was positively associated with positive parenting characteristics and outcomes, while insecure styles were negatively associated (Jones et al., 2014).

Parental motivation, specifically, is an important topic in the study of the association between attachment and parenting. For example, self-report data show that both insecurely attached non-parents (Rholes et al., 1995, 1997; Scharf and Mayseless, 2011; Nathanson and Manohar, 2012) and antenatal couples (Rholes et al., 2006) desire children less. As self-report research is susceptible to common method bias, such as social desirability, it is likely for researchers to overestimate or underestimate the relationships between different attachment styles and parenting.

However, in the field of cognitive neuropsychology, novel methods have been adopted to assess motivation, such as functional magnetic resonance imaging (Strathearn et al., 2008, 2009; Caria et al., 2012) and behavior paradigms (Yamamoto et al., 2009; Parsons et al., 2011; Charles et al., 2013). These studies include images of infants as hedonic stimuli and activation of dopamine-associated reward processing regions, or key pressing to change viewing time of infant images, as indicators of parental motivation. Findings from these researches have revealed that infant images can trigger parental motivation (Strathearn et al., 2008; Yamamoto et al., 2009; Parsons et al., 2011; Caria et al., 2012; Charles et al., 2013) and that different attachment styles create variations in brain activity (Strathearn et al., 2009). Given the above findings, it is evident that a secure attachment style is positively related to parental motivation; however, some limitations remain.

As discussed in our previous research (Cheng et al., 2015b), two problems persist. First, most studies considered attachment style to be relatively stable. This assumption ignores the role major life events play in intimate relationships, various contextual factors that may reshape or influence attachment style, and that these temporary fluctuations are related to meaningful behaviors (Kirkpatrick and Hazan, 1994; Cozzarelli et al., 2003; Davila and Sargent, 2003; Gillath and Shaver, 2007). Second, recent neurocognitive studies have distinguished the brain’s motivational systems of liking and wanting with wanting being divided into representational and evoked responding (Berridge and Robinson, 2003); however, previous research (Yamamoto et al., 2009; Parsons et al., 2011; Charles et al., 2013) has assessed only part of the motivational system such as liking or evoked responding. Additionally, little research has conducted behavioral assessments to examine the relationship between parental motivation and attachment systematically.

In light of these considerations, our previous research directly assessed the link between attachment state and parental motivation using Heerey and Gold’s (2007) behavioral paradigm that is thought to correspond to brain systems (Cheng et al., 2015b). The results of this study indicated that a secure attachment state in adulthood reliably predicted the three positive components of parental motivation by comparing neutral faces of infants and adults.

However, it should be noted that our study (Cheng et al., 2015b), and most studies reported above (Yamamoto et al., 2009; Parsons et al., 2011; Charles et al., 2013), used only neutral infant faces as stimuli. This limitation would narrow the generalizability of the results since parent–child interactions are primarily expressions and voices (Bowlby, 1973). Furthermore, smiling or crying facial expressions are understood to convey the child’s emotional state (Ekman and Fridlund, 1987; Fridlund, 1997) and the need for certain resources from potential caregivers (Trivers, 1974).

A recent empirical study found that adults rated smiling and neutral children as cuter, more adoptable, and less distressing than crying children and viewed smiling videos of children for longer durations than crying children, indicating facial expressions of children elicit motivations for nurturing in non-relatives (Aradhye et al., 2015). Similar findings on the effects of child and infant expressions have been found in neuroimaging studies that show regions of brain activity are differentiated by infant expressions (Strathearn et al., 2008; Lenzi et al., 2009). Overall, it is important to note the impact of infant emotions on adults’ interest in nurturing.

A large number of studies have indicated that attachment is related to several facets of emotion (Shaver and Hazan, 1993; Niedenthal et al., 2002; Maier et al., 2005; Chris Fraley et al., 2006; Strathearn et al., 2009; Donges et al., 2012). For instance, preoccupied participants tend to notice negative emotions (Shaver and Hazan, 1993), whereas those classified as dismissive were apt to avoid negative attachment information (Simpson et al., 1996; Cooper et al., 1998). Moreover, one neuroimaging study reported that individuals with different attachment types had marked differences in brain activation response to their own infant’s crying face (Strathearn et al., 2009). In this research, mothers with secure attachment showed increased activation in reward processing regions, but insecure/dismissive mothers showed greater activation of the anterior insula, a region associated with feelings of unfairness, pain, and disgust.

The studies reviewed above illustrate the ways facial expressions affect adults’ parenting and how different types of attachment activate different responses to various facial expressions. However, there are still two inadequacies. First, the aforementioned behavioral study examined the association between attachment and motivation using only neutral infant faces (Cheng et al., 2015b). Second, although Strathearn et al. (2009) reported different neural responses across attachment styles and facial expressions of infants, a behavior study has yet to be conducted. Therefore, it is essential to extend previous findings by developing a behavioral investigation concerning the effects of infant expressions on parental motivation.

In this study, we primarily aimed to test the link between infant facial expressions and parental motivation as well as the interaction of attachment state and expressions. Specifically, we used Heerey and Gold’s (2007) behavioral paradigm while simultaneously examining how infant facial expressions interact with secure attachment state and subsequently influence parental motivation among non-parents. We hypothesized the following

(1) The specific motivation toward infants will vary according to the types of infant facial expressions.

(2) A positive interaction between attachment state and expression among childless individuals, such that the impact of secure attachment state on parental motivation, will vary according to different infant facial expressions.

## Materials and Methods

### Participants

We recruited 218 healthy undergraduates (118 males, 100 females) from Southwest University. All participants were unmarried and never had children. Participants were aged between 17 and 28 years (*M* = 19.22, *SD* = 1.654) and 94.5% were of Han ethnicity. Each participant was offered 20 Yuan as compensation for their anonymous participation. This research was approved by the Ethics Committee of Southwest University (No. 2014179).

### Measures

This study used the Chinese version (Ma et al., 2012) of the State Adult Attachment Measure (Gillath et al., 2009) to capture individual differences in temporary fluctuations of attachment. The measure is a 21-item self-report Likert scale, ranging from 1 (*strongly disagree*) to 7 (*strongly agree*). It consists of three attachment-state subscales: anxiety, avoidance, and security, and has well-established reliability (internal consistencies for anxiety, avoidance, and security subscales were 0.54, 0.67, and 0.71, respectively) (Ma et al., 2012). Internal consistencies for the present data were anxiety = 0.750, avoidance = 0.676, and security = 0.721. Contrary to the original measure, which had seven items for each subscale, the Chinese version had five items for anxiety and 9 for security. The avoidance subscale was identical for both versions.

Facial stimuli consisted of 48 slides with laughing, neutral, and crying faces (24 adult, 24 infant). Each expression included eight infant, four adult male, and four adult female photos. All slides contained three gray-scale normalized face images of the same expression that were matched for size and luminosity. There were no significant differences between the infant images and adult images in the intensity of each facial expression [laughing: *t*(46) = 0.301, *p* = 0.77; neutral: *t*(46) = 0.755, *p* = 0.45; crying: *t*(46) = 0.214, *p* = 0.83]. These images were taken from the Chinese Infant Affective Face Picture System (Cheng et al., 2015a) and Chinese Affective Face Picture System (Gong et al., 2011).

### Procedure

To ensure test quality, we performed our procedure with up to six participants at a time, based on participant availability. When participants arrived, preliminary information about the study was described and we obtained written informed consent in accordance with the Declaration of Helsinki. Next, participants were instructed to complete the Chinese version (Ma et al., 2012) of the State Adult Attachment Measure. Afterward, to evaluate the wanting and liking components of the motivational system, they were asked to complete a similar computer task to the one utilized in Cheng et al. (2015b) containing three sections, which was programmed by E-Prime, a suite of applications to fulfill the computerized experiment needs. The entire study was approximately 30–40 min in duration.

Hedonic experience (liking) was measured first, which is a self-report measure of the hedonic value of each face. Participants were instructed to rate the extent of pleasure experienced as each slide was presented using the 9-point Self-Assessment Manikin, ranging from 1 (*extremely unpleasurable*) to 9 (*extremely pleasurable*), with 5 (*uncertain*) as the midpoint (Lang et al., 1999).

Representational responding (wanting), the degree to which participants would work to seek or avoid future stimulus exposure, was subsequently measured. Prior to assessment, participants were informed that they would view a slideshow comprising some of the slides they had previously rated. They could press the “n” and “m” keys if they wanted to see a slide again, or the “x” and “z” keys if not. The more “n” and “m” keys they pressed, the more likely the face would be seen again and vice versa for the “x” and “z” keys. This procedure required memory representations because of the absence of each stimulus during the key pressing.

**FIGURE 1:**
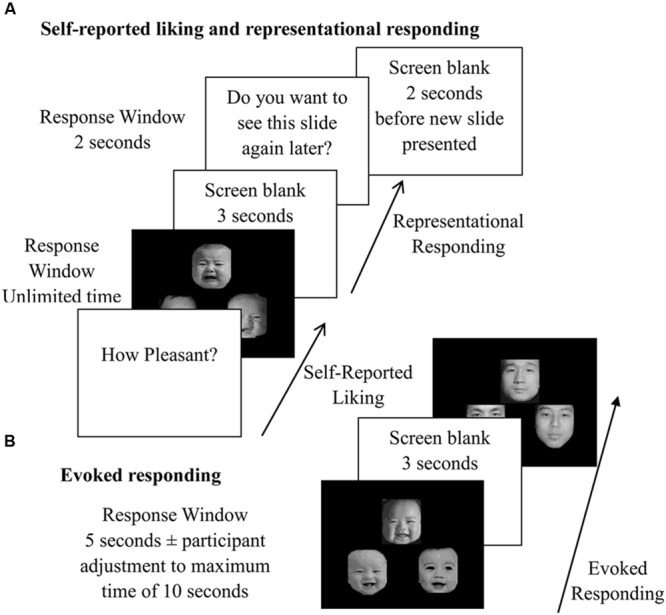
**Study procedure. (A)** Measure of liking interlaced with representational responding, namely, participants rated the pleasantness of one slide and then pressed keys to what extent they want to see the slide again or the opposite. The process is repeated for the rest of slides. **(B)** Participants’ key pressing served to decrease or increase viewing time of the slides being rated.

The evoked responding (consumer behavior) was measured by the degree that the effort was exerted to prolong or reduce exposure to a perceptually available stimulus. Thirty-six of the previously viewed slides (six infant, three adult male, and three adult female of each expression) were displayed to participants. They could repeatedly press the “n” and “m” keys to increase viewing time, or “x” and “z” keys to shorten viewing time. The slides were presented for 5 s in the absence of responding; the maximum time participants could view the slides was 10 s and the minimum view time was 3 s (**Figure 1**).

### Variables and Statistical Methods

The scoring formula was identical to our previous study (Cheng et al., 2015b); that is, we used discrepancies between the responses to infant and adult faces in liking, representational, and evoked responding as the outcome variables, in order to control the effect of human faces. This represented “special motivation toward infants”.

The average score per slide for each infant expression on self-reported liking minus the corresponding score for adult stimulation generated three variables within each participant, that is, the laughing liking, the neutral liking, and the crying liking. These variables represented “special liking toward infants”.

For the special representational responding, we subtracted the total number of presses to avoid undesirable images from the total number of presses to seek desirable images within each category. This indicated a net interest in the category. After that, the net interest for infants minus the net interest for adults was used to indicate, “special representational responding to infants”. The calculation for the special evoked responding to infants is similar to that of special representational responding.

Finally, a random intercepts multilevel regression with emotion nested within participants and a hierarchical regression analysis were used to examine the interaction of emotion and security attachment state and the unique contributions of the security attachment state in predicting the motivation for parenting, respectively. Multilevel models were estimated with Hierarchical Linear and Non-linear Modeling software (using maximum likelihood estimation) and the hierarchical regression analysis was conducted using SPSS 22.

## Results

### Descriptive Statistics

**Table 1** summarizes the average score and standard deviation of the three components of motivational value in different facial expressions.

**Table 1:** Mean and standard deviation of the special motivational value to infant faces.

	Laughing		Neutral		Crying	
	*M*	*SD*	*M*	*SD*	*M*	*SD*
Liking	1.556	1.587	1.989	1.600	0.728	1.098
Representational	107.115	99.466	134.830	99.331	51.775	73.241
Evoked	201.573	171.312	194.675	159.669	61.385	105.310

### Random Intercept Multilevel Model

#### The Special Liking toward Infants

A baseline model without explanatory variables demonstrated that 45.58% of the total variance in the special liking toward infants was accounted for by the variation across participants.

The emotion-level analysis indicated that laughing and crying faces had a significant effect on the special liking compared to neutral faces. Moreover, when compared with neutral expressions, participants showed less special liking for laughing and crying expressions (**Table 2**). There was a significant variation in the variance of the intercept [χ^2^(217) = 1113.90, *p* < 0.001]. The significant variance component for the intercept indicates that individuals’ characteristics may be able to explain special liking levels. This allowed us to explore the effect of secure attachment state on special liking across emotion.

**Table 2:** Summary of a 2-level random intercept multilevel model of the special motivational value to infant faces.

Fixed effects	Liking			Representational			Evoked		
	Estimate	*SE*	*t*	Estimate	*SE*	*t*	Estimate	*SE*	*t*
Condition 1 (neutral)	1.985	0.108	18.389^∗∗^	134.830	6.712	20.088^∗∗^	194.665	10.789	18.042^∗∗^
Condition 2 (crying)	-1.261	0.089	-14.093^∗∗^	-83.055	6.004	-13.834^∗∗^	-133.280	10.002	-13.325^∗∗^
Condition 3 (laughing)	-0.433	0.084	-5.163^∗∗^	-27.716	6.799	-4.076^∗∗^	6.908	11.505	0.600
SAS	0.042	0.015	2.838^∗∗^	3.017	0.810	3.723^∗∗^	3.538	1.357	2.607^∗∗^
Condition 2 × SAS	-0.010	0.013	-0.796	-0.735	0.749	-0.982	-0.956	1.251	-0.764
Condition 3 × SAS	-0.028	0.010	-2.761^∗∗^	-2.635	0.839	-3.141^∗∗^	0.212	1.491	0.142

*SAS, security attachment state; ^∗∗^*p* < 0.01.*

In the second step of the analysis, there was a significant effect for secure attachment state on the special liking, and a significant interaction between secure attachment state and emotion (**Table 2**). Secure attachment state facilitated participants’ special liking to neutral and crying faces, but not to laughing faces.

#### The Special Representational Responding to Infants

Concerning the model of the special liking, a baseline model showed that 32% of the total variance in special representational responding to infants was due to variation across participants. Through emotion-level analysis, we found that laughing and crying faces had a *s*ignificant effect on special representational responding in comparison with neutral faces. Conversely, in comparison with neutral faces, participants had less special representational responding to laughing and crying expressions (**Table 2**). The variation in the intercept variance was significant [χ^2^(217) = 729.28, *p* < 0.001]. In the participant-level analysis, a significant effect for secure attachment state on special representational responding and a significant interaction between secure attachment state and emotion were found (**Table 2**). A secure attachment state fostered participants’ special representational responding under both crying and neutral conditions, but not the laughing condition.

#### The Special Evoked Responding to Infants

Again, 25% of the variance in the special evoked responding to infants was from the variation across participants. The emotion-level analysis indicated that the effects of crying faces versus neutral faces were significant. Participants exhibited less special evoked responding to crying (compared with neutral), but no difference between laughing and neutral faces (**Table 2**). There was still a significant variation in the intercept variance [χ^2^(217) = 636.52, *p* < 0.001]. Then, the participant-level analysis revealed that secure attachment state had a significant effect on special evoked responding (**Table 2**); however, the interaction between secure attachment state and emotion was not significant.

### Hierarchical Regression Analysis

Through the three multilevel models, we detected significant interactions between secure attachment state and emotion for special liking and representational responding. However, the specific prediction of secure attachment state under different facial expressions was not clear. In addition, although no interaction was found in the evoked condition, further analysis was still needed to identify the unique role of secure attachment state across all components of parental motivation by means of controlling for potential confounding variables. Thus, a hierarchical regression was carried out next.

In consideration of the conflicting results regarding sex differences in the motivational salience of infant faces (Parsons et al., 2011; Charles et al., 2013) and the effects of anxiety and avoidance attachment state, we entered these variables in the first step. Then, we entered secure attachment state of adults in the second step.

After controlling for sex, anxiety, and avoidance, secure attachment state consistently positively predicted these special motivational values under the neutral and crying conditions, but not the laughing condition (**Tables 3–5**). The overall pattern of relationships was generally unanimous.

**Table 3:** Summary of hierarchical regression analyses on liking.

Model	Laughing		Neutral		Crying	
	Step 1	Step 2	Step 1	Step 2	Step 1	Step 2
Sex	0.011	-0.003	0.030	-0.011	0.083	0.043
Anxiety	0.085	0.063	0.126	0.063	0.115	0.054
Avoidance	0.114	0.131	0.105	0.155^∗^	-0.026	0.022
Security		0.071		0.209^∗∗^		0.203^∗∗^
*R*^2^	0.025	0.029	0.035	0.072	0.022	0.057

*Standardized regression coefficients are reported. ^∗^*p* < 0.05, ^∗∗^*p* < 0.01.*

**Table 4:** Summary of hierarchical regression analyses on representational responding.

Model	Laughing		Neutral		Crying	
	Step 1	Step 2	Step 1	Step 2	Step 1	Step 2
Sex	-0.440	-0.049	0.086	0.039	0.161^∗^	0.109
Anxiety	0.074	0.065	0.074	0.001	-0.038	-0.119
Avoidance	0.037	0.043	0.046	0.103	0.035	0.098
Security		0.027		0.240^∗∗^		0.265^∗∗^
*R*^2^	0.008	0.009	0.020	0.068	0.022	0.036

*Standardized regression coefficients are reported. ^∗^*p* < 0.05, ^∗∗^*p* < 0.01.*

**Table 5:** Summary of hierarchical regression analyses on evoked responding.

Model	Laughing		Neutral		Crying	
	Step 1	Step 2	Step 1	Step 2	Step 1	Step 2
Sex	0.013	-0.013	-0.001	-0.035	0.144	0.106
Anxiety	0.176^∗^	0.135	0.131	0.078	0.002	-0.059
Avoidance	-0.001	0.031	0.079	0.120	0.062	0.109
Security		0.136		0.172^∗^		0.199^∗∗^
*R*^2^	0.032	0.047	0.027	0.052	0.028	0.061

*Standardized regression coefficients are reported. ^∗^*p* < 0.05, ^∗∗^*p* < 0.01.*

## Discussion

Consistent with our previous study (Cheng et al., 2015b) and others (Yamamoto et al., 2009; Parsons et al., 2011; Charles et al., 2013), we found that the neutral infant faces could stimulate strong parental motivation. This finding is also in line with the Kindchenschema or baby schema in infant faces that was proposed by Lorenz (1943). These schemas have been determined to be intrinsic triggers of motivation for caretaking with neutral infant faces (Glocker et al., 2009).

Based on the emotion-level analysis of multilevel models on liking and representational responding, we found that the effect of baby schema in infant faces varies by different expressions. More specifically, the laughing and crying expressions might undermine the baby schema effects. There is a plausible explanation for this finding—that neutral faces, which have an uncertain and ambiguous expression, may require more cognitive processing than laughing and crying faces, which are more obviously positive or negative. There is some evidence that the judgment of neutral faces is not only influenced by the context in which they are presented (Russell, 1991), but also their preceding context (Prince and Hensley, 1992). Therefore, it is evident that the cognitive processing of neutral faces is complex, especially when the infant faces have unique biological significance as hedonic stimuli. However, this interpretation is only a speculation; therefore, it needs to be verified in future research.

An unexpected result was that no differences were found between visual motivation to neutral and laughing faces from the first level of the model on evoked responding. A possible reason for this finding is that laughing and neutral infant faces elicited strong motivation when these hedonic stimuli were sustained. This may have caused a ceiling effect whereby participants had pressed the keys to the maximum for both neutral and laughing expressions.

According to attachment theory (Bowlby, 1969), human beings are equipped with inborn attachment and caregiving behavioral systems. The caregiving behavioral system is an inherent behavioral system that responds to the needs of dependent others, especially children. If a parent’s caregiving behavior complements his or her child’s attachment behavior, this could improve inclusive fitness or genetic success during evolution. Moreover, parents’ caregiving behavioral systems are affected by their own attachment systems that will provide a solid and stable psychological foundation against the stress of others’ suffering. Consistent with this view, researchers argued that the tendency to attend to others’ distress and provide care could be hindered or suppressed by attachment insecurity (Feeney and Collins, 2001; Gillath et al., 2005).

In this regard, our fundamental purpose was to examine how attachment state interacts with infant facial emotion and influenced parental motivation that consequently contributes to parenting behavior. In this study, only secure attachment state significantly predicted special motivational value (liking and wanting) to neutral (uncertain) and crying (negative), but not smiling (positive), infant expressions consistently after controlling for sex, anxiety, and avoidance attachment state. This finding generally corresponds to studies that indicated that insecure adults desire children less (Rholes et al., 1995, 1997; Scharf and Mayseless, 2011). Results were also consistent with a neuroimaging study showing that mothers with a secure attachment state had increased activation in the reward-processing regions and higher peripheral oxytocin levels to their own crying infants (Strathearn et al., 2009).

In the current study, we found that compared to adult faces, participants with higher secure attachment state showed more positive motivational behaviors to crying infant faces than did those with lower secure attachment state. As Bowlby (1969) proposed, infant crying is considered a highly salient social cue that conveys the need for comfort, protection, and safety. Its goal is considered to be bringing the caregiver back to the infant and eliciting the caretaker’s caregiving behavior system for the purpose of survival. At this time, a high secure attachment state can activate positive models of the self that result in a sense of control and confidence in one’s ability to address others’ suffering (Losoya and Eisenberg, 2001; Mikulincer et al., 2005).

Consistent with this, more self-reported insecurity on attachment style measures were associated with maladaptive responses to distress and difficulty in emotional regulation (Mikulincer and Florian, 1995, 1998; Mikulincer and Shaver, 2007, 2008) as well as less empathy, compassion, and forgiveness (Mikulincer et al., 2001, 2005; Shaver et al., 2009). Accordingly, it is reasonable to assume that insecure mothers who have trouble regulating their own emotions and show less empathy and compassion to the needs of others, may struggle with the challenges and pressure of parenting and have difficulty in responding appropriately to the needs of their children, especially when their children are crying to convey their needs.

Secure attachment state also predicts participants’ visual motivation toward neutral infant faces. As mentioned before, neutral faces need more complex processing because of their ambiguous and uncertain expression. Based on Bowlby’s theory and our findings, we propose that timely and accurate recognition of the infant’s emotion is conducive to improving the parenting quality. As some researchers have suggested, insecure attachment style is related to less maternal sensitivity and failing to interpret child’s social signals (Jones et al., 2014). This may mean that in the case of weak emotional signals, individuals with higher secure attachment state could activate their secure internal working models to show higher parental motivation.

We also found that participants showed high positive motivational behaviors toward laughing infant faces, regardless of attachment state. It is reasonable that laughing is a positive emotion; therefore, there is no threat in this expression. This means that proximity to a laughing baby does not require activation of a secure internal working model. Moreover, the three components of motivational value in different facial expressions means were all positive. This indicates that regardless of expression, participants consistently showed a preference for infants compared with adults.

In summary, this study demonstrates the significant role of secure attachment state in parental motivation toward infants with uncertain and negative emotion. This is consistent with the explanation framework of attachment theory. Further, the results indicate that adults with higher secure attachment state could better cope with infants no matter how the infants behave, while adults with lower secure attachment state are likely to have poor performance when encountering infants with a difficult temperament.

Although our data support our hypotheses, further factors need to be considered when discussing the significance of secure attachment. First, the current study did not control for the effect of attachment dispositions, though the state adult attachment measure could measure both generalized and momentary attachment with high validity and utility (Gillath et al., 2009). This issue should be controlled for in future studies. Second, although the participants in this study were all unmarried and childless, the parenting experience or the time they used to spend with a baby may be another potential confounding variables that requires investigation in future research. Third, the correlational design impedes inferences of causality. Future studies could use longitudinal or priming methods to verify the causality of the above explanation framework.

Despite these limitations, these findings have the potential to inform future research aimed at helping individuals with an insecure attachment state provide positive parenting to difficult infants. Future research should also seek to identify factors that affect one’s secure attachment state and develop intervention programs aimed at improving parental behavior by enhancing parents’ secure attachment state.

## Author Contributions

FD, GC, and DZ contribute to the design of the manuscript, revise it criticaly, approve the final version to be published and agree to be accountable for all aspects of the manuscript in ensuring that questions related to the accurary of any part of the work are appropriately investiegated and resovlved. FD and GC contribute to the acquisition, and interpretation of the data for the manuscript. FD analyzed the data and drafted the manuscript.

## Conflict of Interest Statement

The authors declare that the research was conducted in the absence of any commercial or financial relationships that could be construed as a potential conflict of interest.
